# A case of groin lymphorrhea after a leadless pacemaker implantation

**DOI:** 10.1016/j.hrcr.2025.08.024

**Published:** 2025-08-26

**Authors:** Tsukasa Motoyoshi, Shintaro Yamagami, Tomohiro Sato, Toyoki Okuda, Hirokazu Kondo, Toshihiro Tamura

**Affiliations:** Department of Cardiology, Tenri Hospital, Tenri, Japan

**Keywords:** Atrioventricular block, Complications, Leadless pacemaker, Lymphorrhea, Vascular access

**Key Teaching Points**

• Lymphorrhea from the wound site is a clinically significant complication, because it increases the risk of infection and may occasionally lead to malnutrition.
• Lymphorrhea commonly occurs after a vascular reconstruction or a lymph node dissection, although it is rarely observed after catheter-based procedures. To the best of our knowledge, this is the first case report of lymphorrhea associated with a leadless pacemaker implantation.
• In cases with low-output lymphorrhea, primary closure of the nonhealing wound by suturing may be an effective management option.

## Introduction

Groin lymphorrhea is a common complication after an arterial reconstruction in the groin, abdominal surgery, or inguinal lymph node dissection.^1^^,^^2^ However, there have been few reports of groin lymphorrhea associated with catheter-based procedures.^3^

We report a case of groin lymphorrhea after a leadless pacemaker implantation. To the best of our knowledge, this is the first case report of groin lymphorrhea associated with this procedure.

## Case report

**Figure 1:**
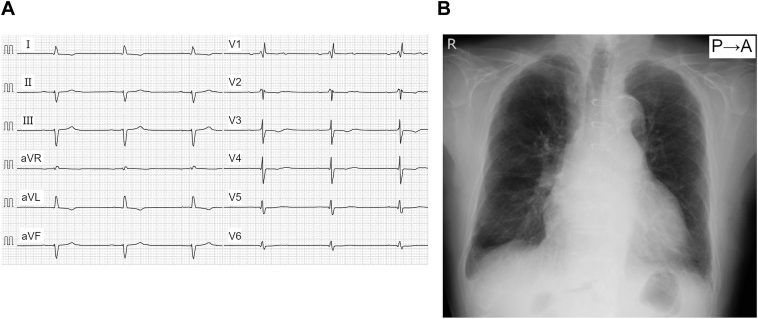
**A:** The 12-lead ECG at presentation, showing 2:1 atrioventricular block with underlying sinus rhythm. **B:** The chest radiograph at presentation demonstrates cardiomegaly, a pleural effusion, and pulmonary edema. ECG = electrocardiogram.

A 94-year-old man was referred from another institution with exertional dyspnea. A 12-lead electrocardiogram (ECG) revealed 2:1 atrioventricular block with underlying sinus rhythm (Figure 1A). A physical examination revealed acute lower extremity edema, and a chest radiograph demonstrated cardiomegaly, a pleural effusion, and pulmonary edema, consistent with a diagnosis of acute heart failure (Figure 1B). He had a history of coronary artery bypass grafting for ischemic heart disease, and transthoracic echocardiography demonstrated a mildly reduced left ventricular systolic function with an ejection fraction of 48%.

The heart failure was considered to be exacerbated by bradycardia owing to atrioventricular block, and a pacemaker implantation was planned. Given the patient’s advanced age and considering the risks of infection and the procedural duration, both leadless and transvenous pacemaker options—including conduction system pacing—were presented to the patient and his family. After shared decision making, a leadless pacemaker was selected.

After the initial management of heart failure, an implantation of a leadless pacemaker (Micra, Medtronic Inc., Minneapolis, MN, USA) was performed on the sixth hospital day. The right femoral vein was punctured under ultrasound guidance, and a 27F Micra introducer sheath was inserted. The leadless pacemaker was successfully implanted without any complications. The puncture site was closed using a figure-of-8 suture technique, and compression with a sandbag was applied to achieve hemostasis.

**Figure 2:**
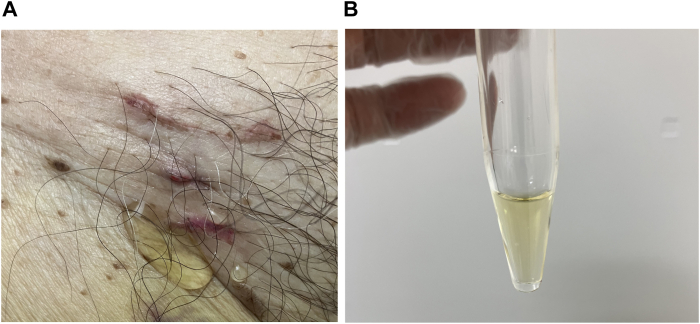
**A:** A serous, clear yellow exudate was noted at the wound site. **B:** Lymphatic leakage was suspected based on the appearance and characteristics of the fluid.

On the following day, the compression was released and the suture was removed without any immediate complications. However, on postoperative day 6, a serous, clear yellow exudate was noted at the wound site (Figure 2A and 2B).

Initially, a surgical site infection was suspected. Therefore, cultures of the wound exudate and blood were obtained, and empirical antibiotic therapy, along with daily wound irrigation, was initiated. Despite these interventions, approximately 20 mL/day of a serous exudate persisted. All cultures returned with negative results, and based on the characteristics of the fluid, lymphatic leakage was suspected. Lymphorrhea was diagnosed after consultation with an experienced surgeon. The lymphatic fluid was observed to originate from a deeper layer than the level of the figure-of-8 suture, suggesting that the leakage was more likely attributable to lymphatic vessel injury associated with the catheter procedure rather than the suture itself.

**Figure 3:**
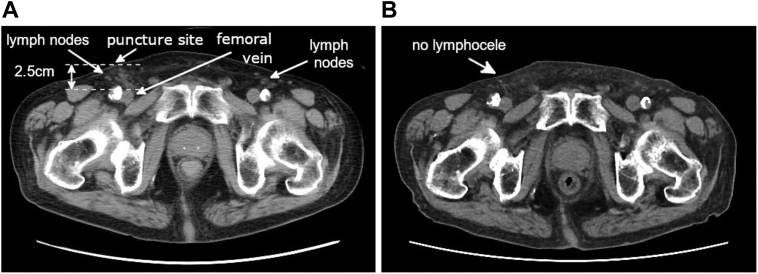
**A:** CT imaging after the leadless pacemaker implantation revealed multiple lymph nodes in both inguinal regions. However, no lymph nodes suitable for intranodal lymphangiography were identified, and there was no evidence of lymph node injury clearly attributable to the puncture. No anatomic abnormalities were observed in the femoral vessels or lymphatic system. The depth from the skin to the femoral vein at the puncture site was 2.5 cm. **B:** Follow-up CT imaging at 6 months postoperatively revealed no evidence of lymphorrhea or a lymphocele. CT = computed tomography.

Compression with a sandbag was applied to treat the lymphorrhea, but it was ineffective. Intranodal lymphangiography and a subsequent embolization were considered, and an interventional radiologist was consulted. However, no accessible lymph nodes suitable for puncture were identified on computed tomography (CT) imaging (Figure 3A), and intranodal lymphangiography was deemed unfeasible.

Ultimately, the nonhealing wound was sutured with nylon, resulting in a successful closure. After this intervention, the external lymphatic leakage resolved, and no lymphocele formation was observed. The patient was discharged in a stable condition. Follow-up CT imaging at 6 months postoperatively revealed no evidence of lymphorrhea or a lymphocele, and there were no signs of edema suggestive of impaired lymphatic drainage (Figure 3B).

## Discussion

Lymphorrhea is typically defined as a persistent leakage of lymphatic fluid through the skin. However, there is no consensus on the drainage volume required to establish this diagnosis.^1^

The incidence of groin lymphorrhea varies across the studies, with reports indicating approximately 5% after a vascular reconstruction,^4^ 0.7% after varicose vein surgery,^5^ and 65% after a lymph node dissection.^6^ Although there have also been reports of groin lymphorrhea associated with catheter-based procedures,^3^ such cases have been rare, and the incidence remains unknown.

Groin lymphorrhea is a clinically important complication, because it increases the risk of a surgical site infection^7^ and may lead to malnutrition in some cases. Reported treatment options include conservative treatment, including local compression and bed rest,^1^^,^^7^ intranodal lymphangiography,^8^ intranodal lymphatic embolization,^9^ doxycycline therapy,^10^ the use of lanreotide,^11^ radiation therapy,^7^^,^^12^ vacuum-assisted closure therapy,^1^^,^^7^ lymphatic ligation,^1^^,^^7^ and muscle flap procedures.^1^^,^^7^

Lymphangiography using lipiodol has been reported to improve the lymphorrhea in 55%–70.3% of patients, indicating that it can serve not only as a diagnostic tool but also as a therapeutic intervention.^8^ In the present case, intranodal lymphangiography was not performed owing to access limitations.

Several strategies may be considered to avoid catheter-related lymphorrhea. It is important to avoid any clearly identifiable lymph nodes under ultrasound guidance. However, in this case, lymphorrhea occurred despite the use of ultrasound and careful avoidance of any visibly apparent lymph nodes.

The lymphatic network in the groin region is located within the femoral triangle—bounded by the inguinal ligament, adductor longus muscle, and sartorius muscle—where the femoral vein passes.^13^ In addition, this network is highly developed, making it difficult to completely avoid injury to the lymphatic vessels during femoral access.

However, catheter-related lymphorrhea differs from that caused by surgical intervention in that the volume of leakage is usually minimal. Therefore, we believe that achieving appropriate wound closure is the most effective strategy to prevent groin lymphorrhea. In the present case, the lymphorrhea originating from an incompletely healed puncture site resolved completely after the wound was sutured and appropriate closure was achieved.

Only a few cases have been reported in which external lymphatic leakage was successfully managed by wound closure alone. This approach may be effective in cases with low-output lymphorrhea.^2^ However, clinicians should be aware that a subcutaneous lymphocele formation may occur in cases with high-output lymphorrhea. Currently, neither a standardized treatment algorithm based on the volume of lymphorrhea nor effective preventive strategies have been established, and further investigation is warranted.^2^

The patient’s body habitus, surgical history, and anatomic abnormalities in the vascular or lymphatic systems may have contributed to the development of groin lymphorrhea. Previous studies have reported aberrant inguinal lymph nodes^14^ and primary lymphedema^15^ as examples of an abnormal lymphatic anatomy, both of which may increase the risk of lymphorrhea.

In the present case, the patient’s body weight was 48 kg and his body mass index was 20.8 kg/m^2^, indicating a normal body habitus. He had a history of coronary artery bypass grafting and colostomy for a sigmoid diverticular perforation, but no history of surgery, interventions, or trauma involving the groin, hip, or pelvis. CT imaging showed no anatomic abnormalities in the femoral vessels, and the depth from the skin to the femoral vein at the puncture site was 2.5 cm. This study also revealed no evidence of lymphadenopathy or other apparent lymphatic anomalies (Figure 3A). However, a definitive evaluation was not possible owing to the unavailability of lymphangiography.

To minimize the risk of lymphorrhea, clinicians should pay close attention to the patient’s body habitus, surgical history, and potential anatomic variations.

## Conclusion

This case represented the first report of lymphorrhea associated with a leadless pacemaker implantation. Although rare, lymphorrhea is an important complication that warrants attention. This report underscores the importance of recognizing and appropriately managing lymphorrhea during such procedures.

## Disclosures

The authors have no conflicts of interest to disclose. The other authors have no conflicts of interest to disclose.
